# Quantification and Classification of Corn and Sunflower Oils as Adulterants in Olive Oil Using Chemometrics and FTIR Spectra

**DOI:** 10.1100/2012/250795

**Published:** 2012-02-01

**Authors:** Abdul Rohman, Y. B. Che Man

**Affiliations:** ^1^Laboratory of Analytical Chemistry, Department of Pharmaceutical Chemistry, Faculty of Pharmacy, Gadjah Mada University, Yogyakarta 55281, Indonesia; ^2^Research Center of Halal Products, Gadjah Mada University, Yogyakarta 55281, Indonesia; ^3^Laboratory of Analysis and Authentication, Halal Products Research Institute, Universiti Putra Malaysia, Serdang 43400, Selangor, Malaysia

## Abstract

Commercially, extra virgin olive oil (EVOO) is subjected to be adulterated with low-price oils having similar color to EVOO. Fourier transform infrared (FTIR) spectroscopy combined with chemometrics has been successfully used for classification and quantification of corn (CO) and sunflower oils (SFOs) in EVOO sets. The combined frequency regions of 3027–3000, 1076–860, and 790–698 cm^−1^ were used for classification and quantification of CO in EVOO; meanwhile, SFO was analyzed using frequency regions of 3025–3000 and 1400–985 cm^−1^. Discriminant analysis can make classification of pure EVOO and EVOO adulterated with CO and SFO with no misclassification reported. The presence of CO in EVOO was determined with the aid of partial least square calibration using FTIR normal spectra. The calibration and validation errors obtained in CO's quantification are 0.404 and 1.13%, respectively. Meanwhile, the first derivative FTIR spectra and PLS calibration model were preferred for quantification of SFO in EVOO with high coefficient of determination (*R* ^2^) and low errors, either in calibration or in validation sample sets.

## 1. Introduction

Extra virgin olive oil (EVOO) is the highest classes of olive oil accounting for an approximately of 10% from olive oil production. Olive oil is among the most important oils used by humans. Olive has contributed to a great economic and social importance for the Mediterranean regions [1]. However, the olive oil is not strictly consumed by Mediterranean people. In the market, olive oil has high price; consequently, olive oil is subjected to be adulterated with other oils having similar color like corn and sunflower oils [2].

From economic reason, some unscrupulous market players may try to add lower-priced plant or nut oils to fresh EVOOs. This action is being unfair to the consumer because incorrect labeling can represent commercial deception [3]. In addition, the adulteration practice may also cause severe health and safety problems, especially to whom having allergy history [4]. Consequently, there is no doubt that the detection of adulteration needs to be addressed in order to ensure the quality of EVOO [5].

Chromatographic-based techniques such as high performance liquid chromatography [6, 7] and gas chromatography [8], especially in combination with mass spectrometer and expensive instruments like NMR spectroscopy [9], are the common analytical technique widely used for detection of EVOO adulteration. However, this technique involves excessive chemical reagents and solvents which are unsafe to human and environmental. For this reason, several efforts have been attempted to detect EVOO adulteration using greener techniques. Such methods are based on vibrational spectroscopic techniques of Raman [5] and infrared [10, 11].

Vibrational spectroscopy can be taken into account as green analytical techniques owing to uselessness of chemical reagents and solvents.

Fourier transform infrared (FTIR) spectroscopy has emerged as powerful and alternative technique for wet and chromatographic methods because little sample preparation is needed, analysis is rapid, and the use of hazardous solvents is minimized. These analytical figures of merit result in time and cost savings and increase the number of analyzed samples [12]. With the aid of chemometric techniques, FTIR spectroscopy has been successfully used for classification and quantification of plant oil adulterants in EVOO. Such adulterants are sesame oil [13] and palm oil [14] quantified with partial least square (PLS) regression, sunflower, corn, soybean, and hazelnut oils using multiple linear regression and linear discriminant analysis [15], corn and sunflower oils using PLS-discriminant analysis [16]. The present study highlights the application of FTIR spectra combined with chemometrics techniques for classification and quantification of corn and sunflower oils after the FTIR spectra are subjected to several spectral treatments.

## 2. Materials and Methods

### 2.1. Materials

Extra virgin olive oil, corn, and sunflower oils were obtained from several supermarkets in Yogyakarta, Indonesia. In order to asses the purity of studied oils, fatty acid composition of oils was determined. The profiles of FA in these oils were compared with those specified in standard Codex [17]. Otherwise specified, all reagents and chemicals used during this study were bought from E. Merck (Darmstat, Germany). The standard of fatty acid methyl esters (C4–C24) was purchased from Sigma Aldrich (USA).

### 2.2. Fatty Acid Analysis

Fatty acid compositions of oil samples were determined with gas chromatography coupled with flame ionization detector (GC-FID). Fatty acid methyl esters (FAMEs) were prepared according to Cocks and van Rede [18]. An approximately 50 mg of oil samples was dissolved in 1.0 mL hexane and added with 0.25 mL sodium methoxide 1 M. The mixture was vortexed and the upper layer containing FAME was transferred to 2 mL vial for a subsequent analysis using gas chromatograph (Agilent Technologies 6890N, Santa Clara, CA). The capillary column used was RESTEX 2330 (0.25 mm internal diameter, 30 m length and 0.2 *μ*m film thickness; Restek Corp, Bellefonte, PA, USA) at a column pressure of 1.03 × 10^5^ Pa. The initial column temperature was 50°C (held for 2 min), then increased to 180° at a rate of 5°C/min, held for 2 min at 180°C, then increased at a rate of 8°C/min to 200°C, and held for 5 min at 200°C. Standard FAME from Sigma was used as authentic samples. The tentative peak identification was done by comparing the relative retention times of samples to those of FAMEs standard. Quantification of FAME was carried out based on internal normalization technique.

### 2.3. Classification

Classification of EVOO and EVOO adulterated with CO, SFO, and the mixture CO-SFO was carried out using discriminant analysis (DA). In this stuy, a set of 20 EVOO samples and 20 EVOO samples adulterated with CO and SFO with concentration ranges of 2.0–50.0% (v/v) was prepared. All samples were scanned with FTIR spectrometer.

### 2.4. Quantification

Quantification of CO and SFO was performed with the aid of multivariate calibrations, namely, principle component regression (PCR) and partial least square (PLS). For analysis of CO in EVOO, a set of 19 calibration samples and 19 validation samples was prepared in neat form comprising of CO in the concentration range of 1.0–50.0% (v/v). Calibration and validation samples of SFO in EVOO were made similar to CO as above. All samples were measured with FTIR spectrometer.

### 2.5. FTIR Spectra Acquisition

All spectra of samples were scanned using FTIR spectrometer Nicolet from Thermo Nicolet Corp., Madison, WI, USA. This instrument was equipped with DTGS detector and KBr/Germanium beam splitter. The operating system used was the OMNIC software (Version 7.0, Thermo Nicolet, Madison, WI, USA). The sampling compartment was Smart Attenuated Total Reflectance kit (Smart ARK, Thermo Electron Corp.) with dimension of 10 × 60 mm. The Smart ARK is an advanced multi-bounce horizontal attenuated reflectance accessory, producing 12 internal reflections with a penetration depth (infrared beam) of 2.0 *μ*m. The accessory was composed of zink selenide (ZnSe) crystal with an aperture angle of 45° and refractive index of 2.4 at 1000 cm^−1^.FTIR spectra were acquired at region of 4000–650 cm^−1^ at co-addition 32 interferograms and resolution of 4 cm^−1^ with strong apodization. These spectra were subtracted against the background of air spectrum. After every scan, a background of new reference air spectrum was taken. The ATR plate was carefully cleaned using soft tissue soaked in hexane and acetone for removing any residues coming from previous samples. The ATR cleanliness was monitored by collecting a background spectrum and compared to the previous one. These spectra were recorded as absorbance values at each data point in triplicate.

### 2.6. Chemometrics

Discriminant analysis and multivariate calibrations employing partial least square (PLS) and principle component regression (PCR) were performed by TQ Analyst Software (Thermo electron Corporation) included in FTIR spectrometer. The difference between actual and calculated values of corn and sunflower oils in calibration model was calculated as root mean square error of calibration (RMSEC). The predictive ability of PLS was assessed by computing root mean square error of prediction (RMSEP) and *R* ^2^ values.

## 3. Results and Discussion

### 3.1. Fatty Acid Composition

It has been explained by some authors that fatty acid composition has been known to affect the exact position and intensity of peaks due to the proportion of saturated and unsaturated fatty acids [19, 20]. SFO and CO exhibited a maximum absorbance at 3009 cm^−1^, while EVOO has maximum peak absorbance at 3006 cm^−1^. The shift of spectral band was attributed from differences in the proportion of oleic acid acyl groups and linoleic and linolenic acyl groups [20]. Fatty acid composition of SFO, CO, and EVOO was shown in Table 1.

### 3.2. FTIR Spectral Analysis

**Figure 1:**
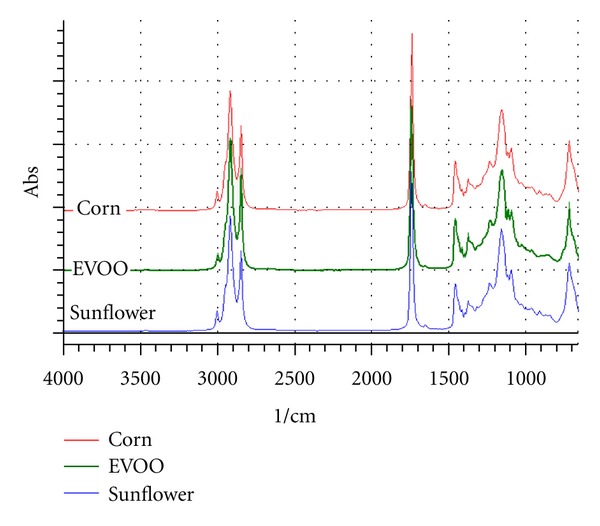
FTIR spectra of extra virgin olive oil, corn oil, and sunflower oil scanned at wavenumbers of 4000–650 1/cm.

The characteristics of mid-infrared spectra for extra virgin olive oil (EVOO), corn oil (CO), and sunflower oil (SFO) are shown in Figure 1. These spectra look very similar and showed a typical characteristic of absorption peaks for common triglyceride, main component composed edible fats and oils. Band at 3007 cm^−1^ is attributed from the stretching vibration of =C–H. Strong band absorptions were observed in the region of 3000–2800 cm^−1^ caused by corresponding to C–H stretching vibrations. The strectching vibrations of methylene (–CH_2_–) and methyl (–CH_3_) groups can be seen at frequencies of 2922 and 2853 cm^−1^, respectively. Methylene and methyl groups are also observed at 1465 cm^−1^ and 1377 cm^−1^ due to their bending vibrations. The large peak around 1740 cm^−1^ is due to C=O double bond stretching vibration. Deformation and bending of C–H and stretching vibration of C–O result in peaks in the 1500–650 cm^−1^ region [21].

The differences among them were clearly small and occurred only in limited regions of the spectra, especially in peak intensities at fingerprint regions (1500–650 cm^−1^) and at 3007 or 3009 cm^−1^. The selection of frequency regions used for analysis was automatically suggested by software; however, analyst should evaluate this region by observing the differences between EVOO and adulterants (CO and SFO).

### 3.3. Classification Analysis

**Figure 2:**
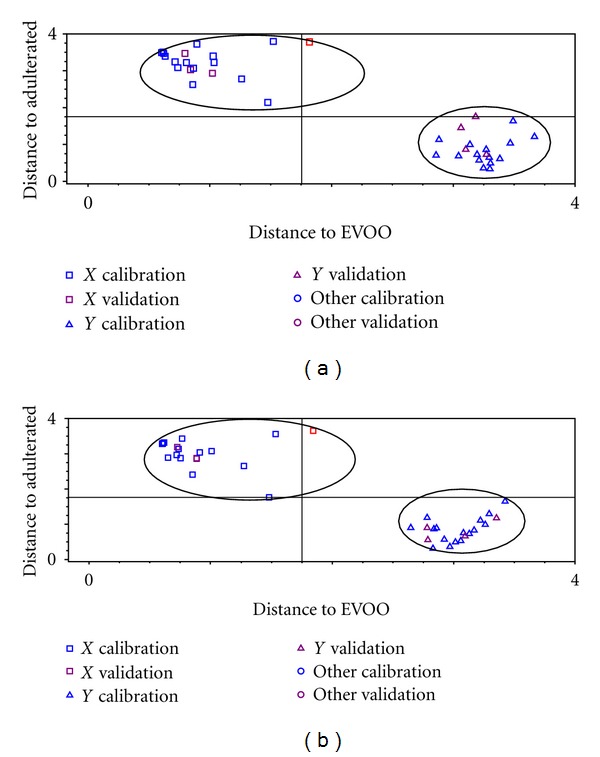
The Coomans plot for classification of EVOO and EVOO adulterated with corn oil (a) and with sunflower oil (b).

Classification of EVOO and EVOO adulterated with SFO and CO was performed with discriminant analysis (DA) using frequency regions of 3027–3000, 1076–860, and 790–698 cm^−1^ (CO) and at frequency regions of 3025–3000 and 1400–985 cm^−1^ for SFO. These frequencies offer good model for classification. In this study, EVOO was adulterated with SFO and CO individually. Figures 2(a) and 2(b) showed the Coomans plot calculated based on the Mahalanobis distance of EVOO adulterated with SFO and CO. The Mahalanobis distance of EVOO mixed with adulterants to EVOO was described in *x-*axis; meanwhile, the distance of EVOO to EVOO added with adulterants was shown in *y-*axis.

The modeled DA can successfully make the classification between EVOO and EVOO adulterated with CO and SFO with no misclassification reported. This means that DA can classify both classes with accuracy level of 100%. Sometimes, the misclassification can occur for some reasons, namely, (i) the close similarity in terms of chemical composition between adulterants and EVOO and (ii) the frequency regions used are not appropriate.

### 3.4. Quantification

Quantification of CO and SFO was carried out with the aid of multivariate calibrations. Two calibration models, namely, partial least square (PLS) and principle component regression (PCR) were used to evaluate the goodness of fit for the relationship between actual value (*x*-axis) and FTIR predicted value (*y*-axis) of CO and SFO in EVOO.  Table 3 compiled the performance of PLS and PCR for quantification of CO in EVOO. Based on Table 3, the first derivative spectra offers the highest coefficient of determination (1.000) and the lowest errors in calibration model expressed as root mean square error in calibration or RMSEC of 0.019% v/v), however, this model shows the high error in prediction model expressed with root mean square error of prediction (RMSEP) of 2.34% v/v. In addition, the number of factors used is to high (8 factors).This means that over-fitting occurs for such model.

**Figure 3:**
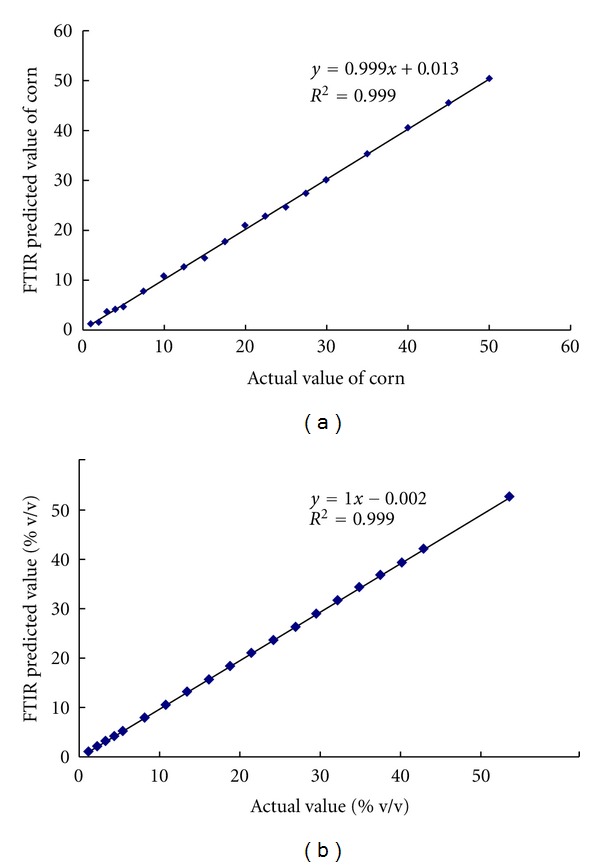
PLS model for the relationship between actual value and FTIR predicted value of corn oil in EVOO using FTIR normal spectra at 790–698, 1076–860, and 3027–3000 cm^−1^; (a) calibration; (b) validation.

Overfitting the regression model is one of the potential disadvantages when using PLS regression [22]. It means that the model generates an optimistic model on the set of data used for calibration (low value of RMSEC), but the model would not perform well on other datasets with similar material, usually used in validation dataset (high value of RMSEP). For this reason, the presence of CO in EVOO was better quantified with PLS using FTIR normal spectra for the reason that low RMSEC value (0.404% v/v) was followed with low error in RMSEP (1.13% v/v).  Figure 3 exhibited the closed relationship between these two parameters either in calibration or validation sample sets.

**Figure 4:**
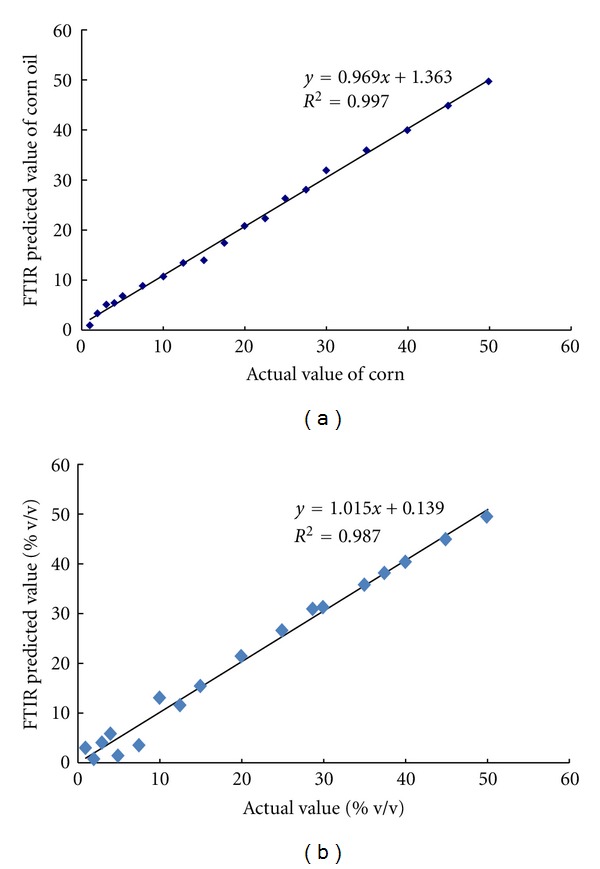
PLS model for relationship between actual value and FTIR predicted value of sunflower oil using FTIR 1st derivative spectra at 3025–3000 and 1400–985 cm^−1^; (a) calibration; (b) validation.

Furthermore, the presence of SFO in EVOO was better quantified using PLS with first derivative spectra (Table 2). Among others, PLS with first derivative spectra gives the reasonable *R* ^2^ either in calibration or in validation and offers the acceptable errors in calibration and validation. Using this model, 8 factors are needed to obtained RMSEC value of 0.034 and RMSEP value of 2.02% v/v. the scatter plot for the relationship between actual value (*x*-axis) and FTIR predicted value (*y*-axis) of SFO in EVOO was revealed in Figure 4. Based on this result, it can be deduced that FTIR spectroscopy with the appropriate selection of calibration model and spectral treatment can facilitate the detection and quantification of CO and SFO as adulterants in EVOO. The developed method is fast and not using the toxic and hazardous solvents and reagents.

## 4. Conclusions

FTIR spectroscopy in combination with discriminant analysis (DA) offers an easy way for classification of EVOO and EVOO adulterated with CO and SFO. DA can accurately classify both classes without any sample misclassification reported. Quantification of CO and SFO as adulterants in EVOO using PLS calibration gives a good calibration and validation model with acceptable errors. The developed method is rapid, free from sample preparation, and not requiring the use of chemicals and reagents; therefore, FTIR technique can be considered as green analytical tools for classification and quantification of EVOO's adulterants.

## Figures and Tables

**Table 1:** FA composition of olive, corn, and sunflower oils.

	The FA composition of studied oils		
	EVOO	CO	SFO
C14 : 0	0.02 ± 0.00	0.06 ± 0.02	0.03 ± 0.01
C16 : 0	10.48 ± 0.12	12.70 ± 0.45	6.81 ± 0.06
C16 : 1	0.66 ± 0.01	0.10 ± 0.01	0.03 ± 0.01
C18 : 0	3.20 ± 0.02	2.01 ± 0.08	3.99 ± 0.15
C18 : 1	71.50 ± 1.15	27.48 ± 0.26	36.86 ± 1.92
C18 : 2	10.65 ± 0.29	53.24 ± 0.92	44.08 ± 0.33
C20 : 0	0.05 ± 0.01	0.09 ± 0.00	0.65 ± 0.00
C18 : 3	0.65 ± 0.01	0.73 ± 0.02	3.68 ± 0.15
C20 : 1	0.46 ± 0.01	0.43 ± 0.01	0.40 ± 0.02
C22 : 0	0.29 ± 0.01	0.20 ± 0.00	0.57 ± 0.01^g^

**Table 2:** The performance of multivariate calibration (MC) and spectral treatments for analysis of corn oil in EVOO.

			Equation		*R* ^2^		RM	RM
MC	Spectra	Factor	Calibration	Prediction	Calibration	Prediction	SEC	SEP
							(% v/v)	(% v/v)
*PLS*	*Normal *	*(3)*	*y* = 0.999*x* + 0.013	*y* = 0.969*x* + 1.363	*0.999*	*0.997*	*0.404*	*1.13*
	1st der	(7)	*y* = 1.000*x* − 0.001	*y* = 0.936*x* + 1.519	1.000	0.977	0.019	2.34
	2nd der	(8)	*y* = 0.999*x* − 0.001	*y* = 0.567*x* + 5.439	1.000	0.534	0.083	10.5

PCR	Normal	(10)	*y* = 0.999*x* + 0.014	*y* = 0.970*x* + 1.136	0.999	0.997	0.394	1.17
	1st der	(10)	*y* = 0.999*x* + 0.012	*y* = 0.931*x* + 1.462	0.999	0.977	0.356	2.33
	2nd der	(10)	*y* = 0.995*x* + 0.095	*y* = 0.567*x* + 5.338	0.995	0.497	1.02	11.0

**Table 3:** The performance of multivariate calibration (MC) and spectral treatments for analysis of sunflower oil in EVOO.

			Equation		*R* ^2^		RM	RM
MC	Spectra	Factor	Calibration	Prediction	Calibration	Prediction	SEC	SEP
							(% v/v)	(% v/v)
PLS	Normal	(8)	*y* = 1.000*x* − 0.001	*y* = 1.097*x* − 0.378	1.000	0.997	0.005	2.34
	*1st der*	*(8)*	*y* = 1.000*x* − 0.002	*y* = 1.015*x* + 0.139	*1.000*	*0.987*	*0.034*	*2.02*
	2nd der	(7)	*y* = 0.999*x* + 0.017	*y* = 0.512*x* + 9.505	0.999	0.580	0.425	10.3

PCR	Normal	(9)	*y* = 0.999*x* + 0.020	*y* = 1.125*x* − 0.447	0.999	0.975	0.426	4.07
	1st der	(9)	*y* = 0.997*x* + 0.067	*y* = 0.958*x* + 1.085	0.997	0.985	0.829	1.98
	2nd der	(9)	*y* = 0.901*x* + 1.976	*y* = 0.083*x* + 11.612	0.901	0.052	4.44	17.2
